# Effects of dietary lycopene on the protection against oxidation of muscle and hepatic tissue in finishing pigs

**DOI:** 10.5713/ajas.19.0133

**Published:** 2019-08-03

**Authors:** Marcelise Regina Fachinello, Eliane Gasparino, Alessandra Nardina Triccia Rigo Monteiro, Cleiton Pagliari Sangali, André Vinicius Sturzenegger Partyka, Paulo Cesar Pozza

**Affiliations:** 1 Department of Animal Science, State University of Maringa, Maringá, Paraná, 87020-900, Brazil; 2 Institut National de la Recherche Agronomique, Agrocampus Ouest, Saint-Gilles, 35590, France; 3 Department of Agronomy, Integrated University Center, Campo Mourão, Paraná, 87300-970, Brazil

**Keywords:** 2,2 Diphenyl 1 Picrylhydrazyl (DPPH), Liver, Meat, Oxidation, Pig, Thiobarbituric Acid Reactive Substances (TBARS)

## Abstract

**Objective:**

The objective of this study was to evaluate the effect of different levels of lycopene supplementation on the carcass traits, meat quality, concentration of lipid oxidation products and antioxidant potential in the meat and liver of finishing barrows and gilts.

**Methods:**

A total of 40 barrows and 40 gilts were allotted in a completely randomized block design, arranged in a 2×5 factorial scheme, consisting of two sexes (barrows and gilts) and five dietary levels of lycopene (0, 12.5, 25.0, 37.5, and 50.0 mg/kg). In addition, four storage times (0, 24, 48, and 72 h), at 4°C, were added to the model to evaluate the *longissimus lumborum* muscle.

**Results:**

An interaction (p = 0.010) was observed between storage periods and dietary lycopene levels. The unfolding of the interaction (lycopene×period) showed a decreasing concentration of malondialdehyde concentration as the dietary lycopene increased, at all storage periods. No interactions (p>0.050) were observed for the 2,2 diphenyl 1 picrylhydrazyl (DPPH) in the pork. However, the percentage of DPPH radical inhibition reduced (p = 0.001) up to 72 h. Additionally, there was a linear increase (p = 0.001) in the capture of DPPH radicals by antioxidants, as the dietary lycopene increased. No interactions were observed (p>0.05) between the evaluated factors in liver. However, lipid oxidation was reduced by supplementing lycopene in pig diets. The capture of the DPPH radical, resulted increase in the antioxidant power exerted by lycopene in the liver (p = 0.001). The concentrations of the thiobarbituric acid reactive substances and DPPH in the liver were affected by sex (p = 0.001).

**Conclusion:**

Dietary supplementation of lycopene reduced the water loss during thawing and was effective in protecting against oxidation of the longissimus lumborum muscle and liver until 72 hours of storage, and the best results were obtained by supplementing with 50.0 mg of lycopene/kg of diet.

## INTRODUCTION

Meat has high amounts of proteins and lipids and is highly susceptible to oxidation. The oxidation processes in meat begins even before the slaughter and intensifies after slaughter and processing and storage of the meat [1]. The lipid oxidation process is one of the main factors related to meat deterioration, affecting nutritional and sensorial characteristics due to the formation of free radicals and unpleasant odors [2]. Pork is highly susceptible to these oxidative changes due to the high content of polyunsaturated fatty acids [3].

Research points to the possibility of attenuating the undesirable chemical substances in meat products through intervention with antioxidants, either through feed supplementation or by directly adding the substances to the processed meat [4]. The use of antioxidants is an effective way to minimize the development of oxidative rancidity, extending the shelf life and nutritional quality of the meat [5].

Lycopene belongs to the carotenoid family and is known to be a powerful antioxidant that protects cells against damage caused by reactive oxygen species because it is highly able to capture singlet oxygen molecules compared with other carotenoids [6]. In addition, lycopene helps to prevent lipid peroxidation by eliminating the initiating radicals [7]. Some studies have already shown improvements in pork quality of pigs fed diets with lycopene. Finishing pigs receiving 20 mg lycopene/kg of diet showed lower malondialdehyde (MDA) concentrations in fresh belly meat [8]. The inclusion of tomato processing by-products in pig diets during a seven weeks period improved the pork softness [9] and the inclusion of 5.0% of this byproduct in the diet increased the oxidative stability of pork, improving its nutritional quality [10]. Due to these antioxidant properties, lycopene supplementation in pig diets could improve the oxidative status of the animal and consequently the pork quality by delaying oxidative processes and improving antioxidant capacity. In the same way, the scientific literature about the effects of dietary supplementation of lycopene extract on the carcass traits and pork quality is lacking. Thus, the objective of this study was to evaluate the effect of dietary levels of lycopene on the carcass traits, meat quality, concentration of lipid oxidation products and antioxidant potential in the meat and liver of finishing barrows and gilts.

## MATERIALS AND METHODS

The experiment was carried out in the swine farm of the State University of Maringá, located in Paraná State (23° 21′S, 52° 04′W and altitude of 564 m), from January 2016 to May 2016. All experimental procedures were previously approved by the Animal Use and Ethics Committee (protocol n° 6570200815).

### Animals, diet and experimental design

Eighty pigs were used, 40 barrows and 40 gilts (Piétrain×Landrace×Large White), with an average initial body weight of 75.04±1.6 kg and final body weight of 100.45±3.69 kg. The pigs were housed in conventional facilities divided into two wings, each consisting of 20 pens (3 m^2^) separated by a central corridor. Each pen had a nipple-type drinker and a semi-automatic feeder, providing *ad libitum* access to feed and water throughout the experimental period. The minimum and maximum temperatures inside the facilities were 19.62°C±2.54°C and 32.11°C±3.2°C, respectively, which were measured by using a data logger (Hobo U10, Bourne, MA, USA) installed in the center of the facilities; data were collected every 30 min during the experimental period.

The animals were allotted in a completely randomized block design, arranged in a 2×5 factorial scheme, with eight replicates per treatment and one animal per experimental unit, and all this information was used to determine each studied parameter. Blocks were set over the time when the pigs, necessary to set at least one block, had attained approximately 75,0 kg of live weight. Blocks and their interactions with other sources of variation were also considered in the statistical model. Treatments consisted of two sexes (barrows and gilts) and five dietary lycopene levels (0, 12.5, 25.0, 37.5, and 50.0 mg of lycopene/kg of diet.). In addition, four storage times (0, 24, 48, and 72 h), at 4°C, were used to evaluate the thiobarbituric acid reactive substances (TBARS) and the 2,2-diphenyl-1-picrylhydrazyl radical (DPPH) of the longissimus lumborum (LL) muscle, providing a 2×5×4 factorial scheme for these last parameters.

The experimental diets (Table 1) were formulated based on corn, soybean meal, minerals, vitamins, and additives, to meet the nutritional requirements proposed by NRC (National Research Council), for each sex. A commercial product containing 10% of a nature-identical, stable formulation of lycopene (Redivivo 10%) with characteristic flavor and odor, was added to the diets, replacing 0, 125, 250, 375, and 500 mg of inert material (fine clean sand), corresponding to the levels of 0, 12.5, 25.0, 37.5, and 50.0 mg of lycopene/kg of diet. The daily lycopene intake was calculated based on the daily feed intake and the percentage of lycopene included in each diet.

### Carcass traits, pork quality and relative organ weight

At the end of the feeding trial, the pigs were fasted for 24 h, weighed to obtain the live weight and then slaughtered in the abattoir of the Maringá State University. The pigs were subjected to electrical stunning (200 W) and then killed by exsanguination, shaved and gutted.

Carcasses were longitudinally middle-divided, weighed and chilled (2°C±1°C for 24 hours) to evaluate the hot carcass weight (HCW), cold carcass weight (CCW), hot carcass yield (HCY), cold carcass yield (CCY), carcass weight loss after cooling, ham yield, backfat thickness (BT), and depth of LL muscle (LL depth) according to the guidelines of Bridi and Silva [11].

The BT and LL depth were measured in the left half of the carcass 24 hours post-mortem using a pachymeter at the insertion of the last thoracic vertebra with the first lumbar, six centimeters from the midline of the carcass (point P2). The lean meat yield (LMY) was predicted by using the equation, as follows: LMY (%) = 60–([BT mm×0.58]+[LL depth mm× 0.10]). The liver and kidneys were weighed, and the relative weights were expressed as a percentage of the HCW. Abdominal fat was also removed and weighed to obtain its relative weight, based on the CCW.

The meat pH was measured in the LL muscle with the aid of a HI 99163 digital portable pH meter (Hanna Instruments, Woonsocket, RI, USA) at the height of the last rib 45 minutes after slaughter (pH45) and after 24 hours of cooling (pH24), according to Bridi and Silva [11].

Three samples (2.5 cm thickness) of the LL muscle of each animal were used for the qualitative evaluations, obtained at the insertion of the last thoracic vertebra with the first lumbar, in the caudal-cranial direction, as described by Bridi and Silva [11]. The first sample was used for color evaluation, the second was used for drip loss (DL), and the third was used to determine the thawing loss (TL), cooking loss (CL) and shear force (SF) [11].

The DL was evaluated according to the procedures described by Boccard et al [12]. To obtain the TL, the frozen samples were weighed, packed in polythene bags, identified and stored in a refrigerator during 24 hours at 4°C to thaw, and the samples were placed in plastic trays, avoiding stacking them. After 24 hours, the samples were withdrawn from the refrigerator, lightly wiped with paper towel and weigheted again. The CL was obtained from the weight difference between the thawed sample and the sample after baking in a preheated oven at 170°C, until reaching an internal temperature of 71°C [11].

Cooked muscle samples were used to determine SF. Six subsamples with a cylindrical shape (1.27 cm in diameter) were longitudinally drawn towards the muscle fibers. The SF was evaluated perpendicular to the orientation of the muscle fibers with the Warner-Bratzler blade adapted in the Stable Mycro Systems TA-XT2i texturometer using a pre-test speed of 5 mm/s in the 2 mm/s test and the 5 mm/s post-test, as recommended for pork.

The color evaluation was performed by six Minolta lightness measurements (L*, a*, and b*) using a portable colorimeter CR-400 Konica Minolta (settings: Illuminant D65, Ramsey, NJ, USA); 0° viewing angle; and 4 auto-average). The components L* (lightness), a* (red-green), and b* (yellow-blue) were expressed using the CIELAB color system.

### Determination of thiobarbituric acid reactive substances

The lipid oxidation evaluation was carried out by determining the substances reactive to thiobarbituric acid. The lipid oxidation evaluation was carried out by determining the reactive substances to thiobarbituric acid. For the samples of hepatic tissue, this evaluation was performed only at hour 0 (slaughter), and for LL muscle the evaluations were performed at hour 0 (slaughter) and at the storage periods of 24, 48, and 72 hours after slaughter (i.e. storage periods of 0, 24, 48, and 72 hours). Samples were collected and stored (liquid nitrogen) until analysis and, after that, were thawed at environmental temperature and monitored until reaching 4°C, packed in plastic film and randomly distributed for cooling storage (4°C) at the different periods.

These procedures were performed according to the improved methodology of Juncher et al [13].

A standard curve was fitted using 1,1,3,3-tetraethoxypropane and distilled water. The absorbance reading was performed at a wavelength of 532 nm, and the results were expressed as mg of malonaldehyde/Eq. kg of tissue.

### Determination of total antioxidant activity by 2,2-diphenyl-1-picrylhydrazyl radicals

The analysis of DPPH radical is based on the capture of the DPPH radical by antioxidants, i.e., the determination of the antioxidant capacity of the sample in reducing the DPPH radical, resulting in a decrease in absorbance (Abs) at 515 nm, according to the methodology described by Brand-Williams et al [14] and Li et al [15], with modifications, and using a 0.06 mM DPPH solution.

The LL muscle samples were analyzed at 0, 24, 48, and 72 hours after refrigeration, and the hepatic tissue samples were evaluated at hour 0 (slaughter). The collection and preparation of the samples proceeded in the same manner as previously described for the TBARS.

The effective concentration that inhibited 50% of the initial concentration of the DPPH radical was determined, with readings at 515 nm. The results were obtained as follows:

% Inhibition DPPH=[(Abs control-Abs sample)/Abs control]×100

### Statistical analysis

The carcass traits, meat quality, relative organ weight, liver concentrations of DPPH and TBARS were statistically evaluated considering a 2×5 factorial scheme, consisting of two sexes (male and female) and five lycopene levels (0, 12.5, 25.0, 37.5, and 50.0 mg/kg of diet). Additionally, the DPPH and TBARS of the LL muscle were statistically evaluated considering a 2×5×4 factorial scheme, consisting of two sexes (males and females), five lycopene levels (0, 12.5, 25.0, 37.5, and 50.0 mg/kg of diet) and four storage times (0, 24, 48, and 72 h).

The UNIVARIATE procedure was used to evaluate the presence of outliers. Subsequently, data were submitted to analysis of variance, and the effects of blocks, sex, lycopene levels and interactions were used in the model to evaluate the carcass traits, meat quality, relative body weight, DPPH and TBARS in the liver, as previously described for the 2×5 factorial scheme. Additionally, the effects of storage period (i.e., 0, 24, 48, and 72 h) and the interactions with the other sources of variation (i.e., sex and lycopene levels) were also used to evaluate the DPPH and TBARS of LL muscle, according to the 2×5×4 factorial scheme previously described.

## RESULTS

### Daily lycopene intake, carcass traits, pork quality, and relative organ weights

To the variable daily lycopene intake an interaction was observed between the sex and dietary lycopene levels (p = 0.006) (Table 2). The unfolding of the interaction revealed an increase in lycopene intake as the dietary lycopene supplementation was increased (p = 0.001), adjusted to fit the equation Ŷ = 2.6342x–0.167 (R^2^ = 0.99). The barrows daily lycopene intake was greater in relation to the gilts (p = 0.001). The carcass traits and relative organ weights were not affected by dietary lycopene levels (p>0.05), and likewise, no interactions were observed (Table 2). Differences between sexes were observed, since barrows presented a higher HCY (p = 0.049), CCY (p = 0.023), BT (p = 0.001) and abdominal fat (p = 0.001) than gilts.

No interactions (p>0.05) between the evaluated factors were observed for the qualitative characteristics of the LL muscle (Table 3). The DL and CL were not affected by lycopene levels (p>0.05), providing a positive effect with lycopene supplementation, since the water losses of the LL muscle did not increase. In the same way, the pH45 and pH24 were also not affected (p>0.05) by lycopene levels (Table 3). The TL decreased in a linear manner (p = 0.024) as lycopene levels increased in the diets, fitting the equation Ŷ = −0.0244x+6.9282 (R^2^ = 0.89; residual standard deviation [RSD] = 0.7710). The SF showed a tendency toward reduction (p = 0.064) according to increasing dietary lycopene levels.

The color of the LL muscle was not affected (p>0.05) by dietary lycopene levels, but sex showed differences for a* (p = 0.001) and b* (p = 0.045), in which the barrows showed higher staining intensity for the red-green and blue-yellow components, respectively.

### Total antioxidant activity by the DPPH radical and lipid oxidation by thiobarbituric acid reactive substances of the LL muscle

**Figure 1:**
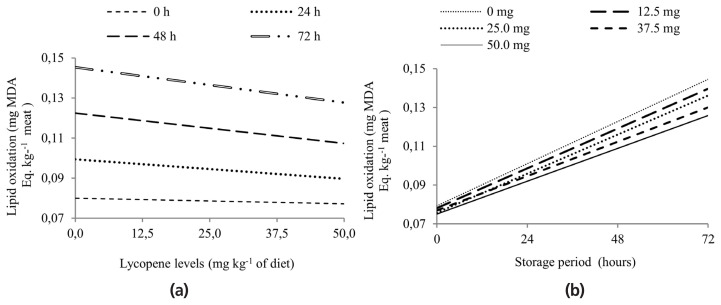
Measurement of lipid oxidation in terms of thiobarbituric acid reactive substances (TBARS). (a) at different storage periods according to lycopene levels, fitting the equations Ŷ = –0.0001x+0.0799 (R_2_ = 0.92; RSD = 0.0016), Ŷ = –0.0002x+0.0994 (R_2_ = 0.85; RSD = 0.0017), Ŷ = –0.0003x+0.1224 (R_2_ = 0.97; RSD = 0.0012), and Ŷ = –0.0003x+0.1453 (R_2_ = 1.00; RSD = 0.0018); for 0, 24, 48, and 72 hours, respectively. (b) at each lycopene level over the storage periods of the longissimus lumborum muscle, fitting the equations Ŷ = 0.0009x+0.0793 (R_2_ = 0.91; RSD = 0.0008), Ŷ = 0.0008x+0.0781 (R_2_ = 0.88; RSD = 0.0029), Ŷ = 0.0008x+0.0757 (R_2_ = 0.88; RSD = 0.0034), Ŷ = 0.0007x+0.0769 (R_2_ = 0.89; RSD = 0.0026), and Ŷ = 0.0007x+0.0750 (R_2_ = 0.89; RSD = 0.0025); for 0; 12.5; 25.0; 37.5; and 50.0 mg of lycopene/kg of diet, respectively.

An interaction was observed (p = 0.006) between the storage periods and dietary lycopene levels (Table 4). The unfolding of the interaction revealed a reduction in lipid oxidation as the dietary lycopene supplementation was increased, for all evaluated periods (Figure 1a), adjusted to fit the equations Ŷ = −0.0001x+0.0799 (R^2^ = 0.92; RSD = 0.0016), Ŷ = −0.0002x+ 0.0994 (R^2^ = 0.85; RSD = 0.0017), Ŷ = −0.0003x+0.1224 (R^2^ = 0.97; RSD = 0.0012), and Ŷ = −0.0003x+0.1453 (R^2^ = 1.00; RSD = 0.0018), for the storage periods of 0, 24, 48, and 72 hours, respectively. When evaluating the interaction within each lycopene level, linear and quadratic equations were fitted over the evaluated periods, but the linear model explained the observed data better, according to the determination coefficients, in which an increase in lipid oxidation showed a direct correlation with the increasing storage time (Figure 1B), fitting the equations Ŷ = 0.0009x+0.0793 (R^2^ = 0.91; RSD = 0.0008 ), Ŷ = 0.0008x+0.0781 (R^2^ = 0.88; RSD = 0.0029), Ŷ = 0.0008x+0.0757 (R^2^ = 0.88; RSD = 0.0034), Ŷ = 0.0007x+ 0.0769 (R^2^ = 0.89; RSD = 0.0026), and Ŷ = 0.0007x+0.0750 (R^2^ = 0.89; RSD = 0.0025), for the levels 0, 12.5, 25.0, 37.5, and 50.0 mg of lycopene/kg of diet, respectively.

No interactions between lycopene levels and sex were observed (p>0.05) for the inhibition of the DPPH radical in the LL muscle. However, the DPPH radical was affected by the storage period and dietary lycopene levels (Table 5). The inhibition of the DPPH radical in the meat was reduced up to 72 h, as described by the equations Ŷ = 0.0594x+39.70 (R^2^ = 0.97; RSD = 0.1677) and Ŷ = 0.0005x^2^–0.0956x+39.99 (R^2^ = 1.00), but the derivation of the quadratic equation overestimated the storage time (95.6 h).

In addition, increasing dietary lycopene levels provided an increase (p = 0.001) in the capture of DPPH radicals by antioxidants in the LL muscle, as described by the equation Ŷ = 0.04279x+36.50 (R^2^ = 0.96; RSD = 0.3351).

### Total antioxidant activity by the DPPH radical and lipid oxidation by the thiobarbituric acid reactive substances of the liver

No interactions were observed (p>0.05) between the evaluated factors (Table 6). Lipid oxidation of the liver was reduced by supplementing lycopene in pig diets, and this response is described by the equations Ŷ = −0.0006x+0.3211 (R^2^ = 0.84; RSD = 0.005) and Ŷ = 0.0000x^2^–0.0014x+0.3261 (R^2^ = 0.98), and the derivation of the quadratic equation provided a level of 34.47 mg of lycopene/kg of diet.

The capture of the DPPH radical by antioxidants in the liver was increased (p = 0.001) (Table 6), resulting in an increase in the antioxidant power exerted by lycopene in the liver due to the increase in dietary supplementation of lycopene (Ŷ = 0.0115x+5.98; R^2^ = 0.84; RSD = 0.1003). The concentrations of TBARS and DPPH in the liver were affected by sex (p = 0.001), in which the gilts showed the lower production of malonaldehyde and greater capture of the DPPH radical by antioxidants than barrows.

## DISCUSSION

### Daily lycopene intake, carcass traits, pork quality and relative organ weights

The inclusion of lycopene in the diet did not influence feed intake, because there was a linear increase in lycopene intake as the levels of lycopene in the diet were increased. In addition, the daily lycopene intake was influenced by sex with the barrows having a higher consumption of lycopene than gilts, this result can be explained by the fact that barrows are less efficient than gilts, requiring a higher feed intake. The carcass traits and relative organ weights were not affected by dietary lycopene levels (Table 2). However, sex showed differences for HCY, CCY, LMY, BT, and abdominal fat. These results may be related to the fact that sex directly affects the body composition of pigs by the action of sex hormones [16].

In this study, the evaluated dietary lycopene levels did not affect the color of the LL muscle, suggesting that lycopene can be added into the diet without affecting the instrumental color parameters of the LL muscle. The same response was observed by Chung et al [17], who evaluated the effect of lycopene on pigs supplemented with by-products of tomato processing and did not observe any effects on the color and pH of pork. However, sex affected the LL muscle color, as barrows presented higher intensities of red than did females, and similar results were observed in another study [18]. Evaluating the factors affecting LL colour [19] observed that the most important factors affecting a* are the content of pigments (haematin) and the metmyoglobin fraction (MetMb), followed by the deoxymyoglobin fraction (Mb) and the internal reflectance (FOP value), but the oxymyoglobin fraction (MbO) did not affected a* value. The intensity of the red (a*) depends mainly on the ratio between the amount of oxidized MetMb and oxygenated MbO2, because MetMb is the less redish of the three myoglobin forms, contributing to a reduction of redness (a*) [20].

The DL and CL did not differ between treatments, suggesting that lycopene supplementation did not result in high water losses of the LL muscle. However, the TL showed a reduction according to increasing levels of dietary lycopene, that is an important economic factor, since a high-water loss provides an unattractive appearance of the meat to the consumer. This reduction of muscle TL as the inclusion of lycopene in the diet increased can be explained by the antioxidants preserving the integrity of the muscle cell membrane, reducing the water loss.

Although the shear force was not affected by increasing lycopene levels in the diet (Table 3), a reducing trend was observed, which may be related to improving the meat tenderness, since antioxidants avoid the oxidation of sarcoplasmic proteins [21] as the main enzymes responsible for *rigor mortis* (μ-calpain and *m*-calpain) have a cysteine residue that can be oxidized. Avoiding the calpain oxidation contributes to a high proteolysis during maturation of the meat, making it soft. The shear force reported in this study shows an extreme softness range, including the maximum value (30.11 N), corroborating the results found in the LL of antioxidant-fed pigs [22].

### Total antioxidant activity by the DPPH radical and lipid oxidation by thiobarbituric acid reactive substances of the LL muscle

Another interesting result of this study was the reduction of lipid oxidation in all of the evaluated storage periods: 0, 24, 48, and 72 hours (Figure 1a) as dietary lycopene supplementation was increased. Lipid oxidation is a process related to the deterioration of fatty acids producing high values of TBARS, which are usually associated with unpleasant meat odor and taste [23] due to the development of a thiobarbituric acid reaction with saturated aldehydes (2-enals and 2-dienals), produced in the finishing phase of lipid oxidation. As in this study, that reaction shows a reduced concentration of malonaldehyde in the LL muscle. An et al [8] also observed that the inclusion of 20 mg of lycopene/kg of diet resulted in lower MDA concentrations in fresh pork belly meats of finishing pigs and observed significant values of lycopene in meat and no change in the fatty acid composition of fresh belly meat. In this way, it is suggested that enhanced oxidative stability of pork meat is due to the incorporation of lycopene, rather than lycopene-induced alteration of fatty acid composition or lipid metabolism.

The oxidation processes tend to increase over the storage days, impairing the shelf life of the meat. In addition, this study showed that lipid oxidation increased over the long storage days for all lycopene levels studied (Figure 1b), which was expected. According to Sindelar et al [24], the minimum lipid oxidation was 0.008 and 0.117 mg MDA/Eq kg for pork, and in this study, values above the minimum lipid oxidation were observed starting at 48 h of storage, only for the samples with the inclusion of 0, 12.5, and 25.0 mg of lycopene. However, starting at 72 h, the LL muscle at all lycopene levels showed values above the minimum suggested by Sindelar et al [24]. According to these results, lycopene was able to delay the oxidation during the studied time (72 hours of pork storage). However, evaluating tomato by-products in one raw pork emulsion, during refrigerated storage for 9 day, Joseph et al [25] observed a lower MDA values for treatments with tomato by-products than the control treatment during 9 days of storage. In the same way, there is extensive evidence indicating the protective role of antioxidants by retarding lipid oxidation and prolonging the lifespan of *in natura* pork meat [2].

This oxidation reaction by lycopene can be explained by the mode of action of the antioxidants, preventing lipid peroxidation due to the elimination of the initiating radicals, or acting as a bond catalyst, such as metal ions, to prevent the initiation of radical generation or by the peroxide decomposition. Thus, it cannot be reconverted into initiator radicals by performing chain breaks to avoid the continuous uptake of hydrogen by active radicals [7]. In the case of lycopene, the antioxidant action is based on its ability to extinguish singlet oxygen (^1^O_2_) and other oxygenated species, resulting in cellular protection against oxidative damage; that is, lycopene intercepts oxidative species before damage occurs. By physically extinguishing ^1^O_2_ with lycopene, the deactivation occurs by transferring the excitation energy of ^1^O_2_ to the lycopene molecule, and this causes the lycopene to reach the triplet state, and the energy of the excited lycopene is dispensed through vibrational interactions, recovering the state of the carotenoid [26] due to its conjugated polyene structure, which is responsible for this reaction, making the lycopene a potent antioxidant.

The efficiency of lycopene as an antioxidant can also be observed from the results of the DPPH radical scavenging test (Table 5), where all meat samples showed a greater capacity to eliminate the DPPH radical due to the increase in dietary lycopene. The antioxidant power of lycopene in the meat increased with its increasing levels in pig diets. No studies evaluating the action lycopene in pigs’ diets, or tomato by-products, on DPPH were reported until now. However, extract of tomato powder [27] displayed antioxidant activity in the DPPH radical-scavenging assay in cooked pork patties during storage at 10°C±1°C in the dark. The DPPH radical elimination assay also revealed that the antioxidant power exerted by lycopene on meat was affected by the storage period (Table 5) because the inhibition of the DPPH radical was reduced over the 72 hours evaluated.

Among the methods used to control lipid oxidation, the use of antioxidants is the most effective, convenient and economical means. In addition to protecting products from deterioration, this additive can also be used for health promotion because of its ability to protect the body against oxidative damage [28]. This protection can be confirmed by the results obtained in this study, such as dietary supplementation of lycopene, in addition to reducing lipid oxidation and increasing the antioxidant power in the meat, with a similar effect on the liver.

### Total antioxidant activity by the DPPH radical and lipid oxidation by thiobarbituric acid in the liver

Lipid oxidation of the liver was reduced by the supplementation of lycopene in the pig diet, in which supplementation of 34.47 mg of lycopene/kg of diet resulted in a lower oxidation of the hepatic tissue (Table 6), showing that lycopene can be a potent antioxidant in eliminating free radicals *in vivo*. Due to its polyene structure, lycopene provides an electron-rich system and is an eligible target for electrophilic reagents. Thus, lycopene shows high reactivity against free radicals and oxygen [26]. Similar results were observed by Sun et al [6], reporting that dietary supplementation of lycopene reduced the malonaldehyde content in the liver.

The capture of the DPPH radical increased linearly in the liver of the pigs (Table 6) with the increasing inclusion of lycopene in the diet, resulting in an increase in the total antioxidant power of 7.08%. Similar values were found by Joseph et al [25], showing that pig liver DPPH was 9.90%. In addition, some studies suggest a dose-dependent effect on radical removal of DPPH by antioxidants [29]. Lycopene of diet-origin has the liver as a main destination [6], probably this is the explanation of the lower concentration of MDA and greater capture of the radical DPPH in the liver of finishing pigs, since lycopene acts by intercepting oxidant species before tissue damage occurs [26]. The TBARS and DPPH in the liver were affected by sex, and gilts presented a lower lipid oxidation and higher capture of the DPPH by antioxidants than barrows. Females appear to be less susceptible to oxidative damage due to the higher genetic expression of antioxidants and to the lower oxidative damage of mitochondria [30]. In addition, there is evidence of the strong antioxidant properties of estrogen and differences in nicotinamide adenine dinucleotide phosphate-oxidase activity [31]. Liu et al [32] reported higher levels of MDA in male rats, and this difference was attributed to sex because of the effects of sex hormones.

This effectiveness of lycopene in protecting LL meat and liver of pigs against oxidation (Tables 4, 5, and 6) is of great importance because lipid oxidation is a process in which unsaturated fatty acids react with oxygen and free radicals, through a chain reaction mechanism giving rise to the formation of lipid hydroperoxides and other by-products, such as aldehydes that are responsible for rancidity and spoilage of food [33]. Pork and its products are highly sensitive to lipid oxidation [3], which can mainly affect meat quality attributes such as color, taste, texture and nutritional values [2], which are determining factors for the consumer.

## CONCLUSION

The inclusion of lycopene in the diet of barrows and gilts reduced the liquid loss in thawing and was effective in the protection against oxidation of LL muscle and liver until 72 hours of storage, and the best results were obtained when 50.0 mg of lycopene/kg of diet was supplemented.

## Figures and Tables

**Table 1:** Ingredients, chemical and energetic composition of basal diet

Items	Barrows	Gilts
Ingredients (%)		
Corn	87.14	83.89
Soybean meal 45%	9.61	12.47
Soybean oil	0.740	0.950
Dicalcium phosphate	0.785	0.973
Limestone	0.553	0.521
Salt	0.203	0.203
L-lysine HCl 78.4%	0.373	0.390
L-threonine 98.5%	0.068	0.072
DL-methionine 99%	0.036	0.053
L-tryptophan 98%	0.022	0.017
Growth promoter1)	0.015	0.015
Vitamin and mineral supplement2)	0.400	0.400
Lycopene extract3)	0.000	0.000
Inert4)	0.050	0.050
Total	100.00	100.00
Composition		
Metabolizable energy (Mcal/kg)	3.30	3.30
Nitrogen (%)	1.86	2.03
Total calcium (%)	0.45	0.49
Available phosphorus (%)	0.23	0.26
Potassium (%)	0.42	0.47
Sodium (%)	0.10	0.10
Chlorine (%)	0.18	0.18
SID lysine (%)	0.690	0.770
SID met+cis (%)	0.400	0.440
SID threonine (%)	0.440	0.480
SID tryptophan (%)	0.120	0.130

SID, standardized ileal digestibility.

1) Enramycin 0.015%.

2) Content kg^−1^ diet; vit. A, 30,000 UI; vit. D_3_, 5,000 UI; vit. E, 120 UI; vit. K, 5 mg; vit. B_12_, 120 mcg; niacin, 150 mg; calcium pantothenate, 75 mg; folic acid, 8 mg; choline chloride, 0.48 g; iron, 350 mg; copper, 15 mg; manganese, 250 mg; Zinc, 0.75 g; iodine, 10 mg; selenium, 3 mg.

3) Commercial product, containing 10% of a nature-identical, stable formulation of lycopene (Redivivo 10%) with characteristic flavor and odor, replacing the inert of the basal diets.

4) Fine clean sand.

**Table 2:** Daily lycopene intake, carcass traits and relative organ weights of barrows and gilts, from 75 to 100 kg, fed diets containing different lycopene levels

Items	Barrows					Gilts					SEM	p-value			
		
	Lycopene (mg/kg of diet)					Lycopene (mg/kg of diet)						Sex×lycopene	Sex	Lycopene	
		
	0	12.5	25.0	37.5	50.0	0	12.5	25.0	37.5	50.0				Lin1)	Qua2)
Daily lycopene intake (mg)	0.00	34.61	71.72	101.11	140.79	0.00	29.85	61.21	94.85	122.73	14.638	0.006	0.001	0.0013)	0.618
Hot carcass weight (kg)	78.88	78.80	81.55	79.85	81.32	79.98	79.08	77.64	81.41	79.98	0.329	0.279	0.557	0.160	0.712
Hot carcass yield (%)	82.17	82.75	83.33	83.16	83.28	76.99	76.53	75.04	78.75	77.33	0.098	0.524	0.049	0.375	0.641
Cold carcass weight (kg)	76.40	76.42	78.53	77.47	78.80	82.64	82.15	81.97	82.75	82.07	0.344	0.400	0.454	0.124	0.705
Cold carcass yield (%)	79.59	80.24	80.25	80.68	80.72	79.54	79.50	79.22	79.91	79.36	0.126	0.855	0.023	0.265	0.742
Carcass weight loss in cooling (%)	3.13	3.03	3.69	2.99	3.07	3.75	3.22	3.35	3.28	3.30	0.083	0.558	0.293	0.371	0.903
Ham yield (%)	29.27	29.30	29.09	29.27	29.63	30.23	29.84	29.60	29.21	30.13	0.126	0.906	0.117	0.944	0.155
Lean meat yield (%)	57.54	56.25	58.24	56.66	58.04	59.21	59.39	59.10	58.93	58.81	0.212	0.646	0.004	0.784	0.065
Backfat thickness (cm)	1.56	1.80	1.46	1.65	1.53	1.24	1.19	1.22	1.28	1.24	0.035	0.767	0.004	0.627	0.650
LL depth (cm)	6.07	5.93	6.35	6.25	6.11	6.40	6.31	6.20	6.34	5.93	0.052	0.569	0.451	0.546	0.361
Relative weight of liver (%)	1.739	1.716	1.636	1.735	1.603	1.694	1.711	1.685	1.657	1.759	0.010	0.361	0.717	0.484	0.669
Relative weight of kidney (%)	0.393	0.400	0.381	0.383	0.367	0.393	0.439	0.366	0.422	0.401	0.008	0.713	0.203	0.519	0.817
Relative weight of abdominal fat (%)	1.833	1.739	1.630	1.872	1.992	1.312	1.346	1.401	1.414	1.525	0.044	0.524	0.001	0.055	0.122

The eighty samples used.

SEM, standard error of the mean; LL depth, depth of *longissimus lumborum*.

1) Linear effect of lycopene levels.

2) Quadratic effect of lycopene levels.

3) Ŷ = 2.6342x–0.167 (R^2^ = 0.99).

**Table 3:** Qualitative characteristics of the *longissimus lumborum* muscle of barrows and gilts, from 75 to 100 kg, fed diets containing different lycopene levels

Items	Barrows					Gilts					SEM	p-value			
		
	Lycopene (mg/kg of diet)					Lycopene (mg/kg of diet)						Sex×lycopene	Sex	Lycopene	
		
	0	12.5	25.0	37.5	50.0	0	12.5	25.0	37.5	50.0				Lin1)	Qua2)
pH45	6.34	6.31	6.36	6.30	6.30	6.28	6.20	6.53	6.41	6.43	0.031	0.745	0.627	0.506	0.689
pH24	5.75	5.82	5.71	5.79	5.76	5.75	5.72	5.90	5.73	5.85	0.012	0.838	0.595	0.474	0.838
Drip loss (%)	5.35	5.64	4.93	5.08	4.75	5.14	4.89	4.92	3.93	4.47	0.152	0.915	0.269	0.199	0.950
Minolta L^*^	56.57	55.70	57.77	58.33	59.04	57.21	57.06	56.43	56.83	54.84	0.193	0.880	0.147	0.613	0.880
Minolta a^*^	6.50	6.26	7.14	6.54	7.02	5.27	5.41	5.31	5.56	5.61	0.093	0.524	0.001	0.258	0.975
Minolta b^*^	3.34	3.13	3.85	3.71	3.37	3.22	3.36	2.99	3.17	2.55	0.085	0.388	0.045	0.531	0.232
Thawing loss (%)3)	7.28	6.65	5.83	5.81	5.41	6.77	6.70	6.20	6.34	6.19	0.229	0.846	0.524	0.024	0.571
Cooking loss (%)	27.48	26.53	26.57	24.89	25.94	24.51	24.72	24.34	25.31	24.33	0.165	0.806	0.111	0.548	0.909
Shear force (N)	30.11	29.91	29.22	26.87	25.89	28.15	28.05	27.65	27.26	27.36	0.193	0.655	0.592	0.064	0.635

The eighty samples used.

SEM, standard error of the mean. RSD, residual standard deviation.

1) Linear effect of lycopene levels.

2) Quadratic effect of lycopene levels.

3) Ŷ = −0.0244x+6.9282 (R^2^ = 0.89; RSD, 0.7710); the independent variable “x” represents the lycopene levels in the fitted equations.

**Table 4:** Effects of dietary levels of lycopene for barrows and gilts (75 to 100 kg) on lipid oxidation (mg MDA Eq/kg) of the *longissimus lumborum* muscle at different storage periods

Periods (h)	Barrows					Gilts					SEM
	
	Lycopene (mg/kg of diet)					Lycopene (mg/kg of diet)					
	
	0	12.5	25.0	37.5	50.0	0	12.5	25.0	37.5	50.0	
0	0.0803	0.0787	0.0775	0.0769	0.0762	0.0797	0.0804	0.0784	0.0791	0.0783	0.001
24	0.1009	0.0985	0.0923	0.0936	0.0869	0.0993	0.0957	0.0917	0.0942	0.0920	0.002
48	0.1237	0.1184	0.1175	0.1101	0.1073	0.1210	0.1169	0.1165	0.1108	0.1068	0.003
72	0.1470	0.1431	0.1401	0.1339	0.1289	0.1431	0.1393	0.1335	0.1292	0.1270	0.003

The eighty samples used.

MDA, malondialdehyde; SEM, standard error of the mean.

Statistical significances: lycopene, linear effect (p = 0.001); period, linear (p = 0.001) and quadratic (p = 0.001) effects; no effects for sex (p = 0.213) period×lycopene (p = 0.006) sex×period (p = 0.099), sex×lycopene (p = 0.635), and period×sex×lycopene (p = 0.704).

**Table 5:** Effects of dietary levels of lycopene for barrows and gilts (75 to 100 kg) on the % of inhibition of DPPH radicals in the *longissimus lumborum* muscle

Periods (h)	Barrows					Gilts					SEM
	
	Lycopene (mg/kg of diet)					Lycopene (mg/kg of diet)					
	
	0	12.5	25.0	37.5	50.0	0	12.5	25.0	37.5	50.0	
0	38.771	39.107	39.012	40.768	40.733	39.343	39.809	40.284	41.284	40.923	0.407
24	36.675	37.764	38.274	37.451	38.989	37.227	37.606	38.219	38.304	39.311	0.358
48	34.938	36.375	36.541	37.011	37.083	35.045	36.578	36.958	37.503	37.788	0.421
72	34.349	35.816	36.181	36.401	36.549	34.063	35.177	35.213	36.532	36.919	0.438

The eighty samples used.

DPPH, radical 2,2-diphenyl-1-picrylhydrazide; SEM, standard error of the mean.

Statistical significances: lycopene, linear (p = 0.001) and quadratic (0.362) effects; period, linear (p = 0.001) and quadratic (p = 0.018) effects; no effects for sex (p = 0.278) period×lycopene (p = 0.962) sex×period (p = 0.587), sex×lycopene (p = 0.950), and period×sex×lycopene (p = 0.507).

**Table 6:** Effects of dietary levels of lycopene for barrows and gilts (75 to 100 kg) on the lipid oxidation by TBARS methodology (mg MDA/Eq. kg) and % of inhibition of DPPH radicals of the liver

Items	Barrows					Gilts					SEM	p-value			
		
	Lycopene (mg/kg of diet)					Lycopene (mg/kg of diet)						Sex×lycopene	Sex	Lycopene	
		
	0	12.5	25.0	37.5	50.0	0	12.5	25.0	37.5	50.0				Lin1)	Qua2)
TBARS3)	0.3302	0.3205	0.3094	0.2996	0.3027	0.3197	0.3069	0.2939	0.2894	0.2938	0.006	0.984	0.004	0.001	0.038
DPPH4)	5.8202	5.9244	6.2461	6.3839	6.3642	6.0372	6.2588	6.5662	6.5934	6.5325	0.111	0.958	0.003	0.001	0.068

The eighty samples used.

TBARS, thiobarbituric acid reactive substances; MDA, malondialdehyde; DPPH, radical 2,2-diphenyl-1-picrylhydrazide; SEM, standard error of the mean; RSD, residual standard deviation.

1) Linear effect of lycopene levels.

2) Quadratic effect of lycopene levels.

3) Ŷ = −0.0006x+0.3211 (R^2^ = 0.84; RSD = 0.0051). Ŷ = 0.00002x^2^–0.0014x+0.3261 (R^2^ = 0.98).

4) Ŷ = 0.0115x+5.98 (R^2^ = 0.84; RSD = 0.1003); The independent variable “x” represents the lycopene levels in the fitted equations.
